# Straw Addition Enhances Crop Yield, Soil Aggregation, and Soil Microorganisms in a 14-Year Wheat–Rice Rotation System in Central China

**DOI:** 10.3390/plants13070985

**Published:** 2024-03-29

**Authors:** Bo Liu, Hao Xia, Chaoqiang Jiang, Cuncang Jiang, Muhammad Riaz, Li Yang, Yunfeng Chen, Xianpeng Fan, Zhiyi Zhang, Xiaoli Duan, Maoqian Wu, Xiange Xia

**Affiliations:** 1 Key Laboratory of Fertilization from Agricultural Wastes, Ministry of Agriculture and Rural Affairs, National Station for Qianjiang Agro-Environment, Institute of Plant Protection and Soil Fertilizer, Hubei Academy of Agricultural Sciences, Wuhan 430064, Chinachen971314@163.com (Y.C.); said12170@163.com (M.W.); 2 Tobacco Research Institute, Anhui Academy of Agricultural Sciences (AAAS), Hefei 230001, China; 3 Microelement Research Center, College of Resources and Environment, Huazhong Agricultural University, Wuhan 430070, China; 4 College of Resources and Environment, Zhongkai University of Agriculture and Engineering, Guangzhou 510225, China; riaz1480@hotmail.com

**Keywords:** straw application, chemical fertilizer, soil fertility soil aggregate, microbial community

## Abstract

Straw return utilizes waste resources to reduce the use of chemical fertilizers worldwide. However, information is still lacking on the relative impact of straw return on soil fertility, the nutrient composition of different soil aggregates, and soil microbial communities. Therefore, this study aimed to understand the effects of different management practices on the crop yield, soil fertility, and soil community composition in a 14-year wheat–rice rotation system. The treatments included a control (without fertilizer and straw addition), chemical fertilization (NPK), straw return without fertilizer (S), and straw addition with chemical fertilizer (NPKS). The results showed that NPKS improved the wheat and rice yield by 185.12% and 88.02%, respectively, compared to the CK treatment. Additionally, compared to the CK treatment, the N, P, and K contents of the wheat stem were increased by 39.02%, 125%, and 20.23% under the NPKS treatment. Compared to the CK treatment, SOM, TN, TP, AN, AP, AK, CEC, AFe, AMn, ACu, and AZn were increased by 49.12%, 32.62%, 35.06%, 22.89%, 129.36%, 48.34%, 13.40%, 133.95%, 58.98%, 18.26% and 33.33% under the NPKS treatment, respectively. Moreover, straw addition promoted the creation and stabilization of macro-aggregates in crop soils. The relative abundance of macro-aggregates (0.25–2 mm) increased from 37.49% to 52.97%. Straw addition was associated with a higher proportion of aromatic and carbonyl carbon groups in the soil, which, in turn, promoted the formation of macro-aggregates. Redundancy analysis showed that straw return significantly increased the microbial community diversity. These findings demonstrate that straw addition together with chemical fertilizer could increase the crop yield by improving soil fertility, soil aggregate stability, and the diversity of fungi.

## 1. Introduction

Soil aggregates can affect many physical and chemical processes in the soil, including the recycling of nutrients, soil properties, and soil erosion [1]. The stability of soil aggregates is influenced by soil properties such as the soil texture, organic carbon, and pH, which, in turn, contribute to the formation and stability of the soil structure [2,3]. In the topsoil, 90% of soil organic carbon (SOC) is stored in soil aggregates [4]. SOC is an important indicator of soil health, and its content can influence the soil aggregate composition and its long-term stability [2,5,6]. Soil aggregates also play a crucial role in regulating the decomposition of SOC [7], while soil aggregates generally promote soil carbon sequestration by providing a physical barrier between organic carbon and microbial decomposers [8,9]). Research has shown that soil aggregates are complex, and various environmental variables, including the soil CEC, porosity, moisture, and inorganic C content, influence the soil aggregate size and stability [10]. This is critical, as microorganisms in the soil directly impact nutrient turnover, especially in the decomposition of organic matter [11,12].

Globally, approximately 6.2 billion tons of straw are produced every year [13]. China alone produced 850 million tons of straw in 2017 [14]. Considerable research has been conducted to reuse the residues from this production, rather than simply burning straw waste [15]. Consequently, research has prioritized making full use of crop waste to reduce reliance on chemical fertilizers, improve soil fertility, and develop sustainable agriculture [16,17].

Crop straw residues contain a variety of nutrients that can indirectly release a large amount of nutrients into the soil and gradually change the soil environment [18]. Researchers have found that straw return to the field generally improves soil conditions by enhancing the soil structure, porosity, and bulk density [19]. Moreover, straw return can not only improve the soil environment but also affect the carbon cycle [20]. Wang and Zhang [15] found that the application of waste residues alters the soil C/N ratio, and increases soil organic carbon mineralization. The input of organic matter, such as straw, can promote the creation of macroaggregates and significantly enhance the relative SOC content [21,22]. Soil aggregates also provide a habitat for soil microbes, thus directly affecting the soil microbial biomass, microbial diversity, and community structure [23]. Microorganisms play a critical role in mineralizing and cycling soil nutrients, which is indispensable in the process of straw decomposition [24]. Microorganisms release organic matter, which can create aggregates [25]. Large aggregates are formed by fungi, particularly arbuscular mycorrhizal fungi, through the production of hyphae and root hairs [21]. Moreover, the easily decomposed carbohydrates and amines in straw can be utilized by microorganisms, influencing the structure and diversity of the microbe community [26].

Research has been conducted on the mineralization of SOC as a result of straw return [18]. However, little research has explored the effect of straw return on organic carbon, the structure of different soil aggregates, and the soil microbial community after long-term application. Therefore, we explored how long-term fertilization and straw return influence crop yield, soil fertility, soil microbial activity, and the soil aggregate composition. We aim to provide a theoretical basis for the connection between the management and stability of the soil organic carbon pool and carbon sequestration potential in long-term rice–wheat rotation operations.

## 2. Materials and Methods

### 2.1. Experimental Site

The field experiment was located in Qianjiang (112°37′15.4″ E, 30°22′55.1″ N), Hubei province, China (established in 2005 and harvested in 2019). The soil was flavor-aquic soil developed from the parent material of river alluvium with a pH of 7.10, a soil organic matter (SOM) content of 20.62 g kg^−1^, a total nitrogen (TN) content of 1.53 g kg^−1^, a total phosphorus (TP) content of 0.88 g kg^−1^, an available nitrogen (AN) content of 121.30 mg kg^−1^, an available phosphorus (AP) content of 19.16 mg kg^−1^, an available potassium (AK) content of 59.10 mg kg^−1^ and a bulk density of 1.20 g cm^−3^. The wheat straw was taken from fields and had total N, P, and K contents of 0.54%, 0.09%, and 1.25%, respectively, as reported in our previous study [18].

### 2.2. Field Fertilization Experiment Design

Four different treatments were used, i.e., a control (CK, without chemical fertilizer and straw); NPK (chemical fertilizer alone); S (straw return alone); and NPKS (chemical fertilizers with straw return). In the rice season, 150 kg·hm^−2^ of N, 39 kg·hm^−2^ of P, and 75 kg·hm^−2^ of K fertilizers were applied. In the wheat season, 120 kg·hm^−2^ of N, 33 kg·hm^−2^ of P, and 50 kg·hm^−2^ of K fertilizers were applied. In addition, 6000 kg hm^−2^ of crushed wheat straw was applied to the field by crushing and covering. Each treatment was replicated four times, for a total of 16. Each plot in the field experiments covered 20 m^2^ in rice/wheat rotation. Rice was transplanted in early June every year and harvested at the end of September. Wheat was sown in early October and harvested in early May.

### 2.3. Sample Collection and Determination

#### 2.3.1. Soil Samples

In each plot, soil samples were collected from the topsoil (0–20 cm). The air-dried soil samples were sieved (20-mesh and 100-mesh) for the determination of the soil’s chemical properties. The soil pH was measured using a pH meter (2.5:1 water/soil ratio) and the SOC was determined using a potassium dichromate oxidation–external heating method. The soil’s total nitrogen (TN) and total phosphorus (TP) were measured with the Kjeldahl method and the molybdenum blue method, respectively. The available nitrogen (AN), available phosphorus (AP), and available potassium (AK) were determined using alkali hydrolysis diffusion, NaHCO_3_ extraction molybdenum–antimony colorimetry, and CH_3_COONH_4_ extraction–flame photometry, respectively. The soil exchange calcium (ExCa) and exchange magnesium (ExMg) were measured through ammonium acetate extraction–atomic absorption spectrophotometry. The available soil iron (AFe), manganese (AMn), copper (ACu), and zinc (AZn) were determined with inductively coupled plasma spectroscopy (ICP-OES).

The soil samples were separated by size using sieves (0.053, 0.25, 2 mm) and were shaken for 30 min (soil aggregate analyzer, DIK-2012, Daiki Rika Kogyo Co., Ltd., Akagidai, Japan) [27]. The ratios of each aggregate group, namely the >2, 0.25–2, 0.053–0.25, and <0.053 mm fractions, were analyzed.

The soil aggregate stability was described by the mean weight diameter (MWD), geometric mean diameter (GMD), number of aggregates >0.25 mm (R_0.25_), and fractal dimension index (D) [28]. The soil porosity was measured with the loop-knife method, as described by Liu et al. [29].

For ^13^C-NMR analysis, the soil samples were pretreated with HF (10%) to remove Fe^3+^ and Mn^2+^. During pretreatment, 5 g of dried soil sample was placed into a 100 mL tube containing 50 mL of 10% HF solution. Each sample was then rinsed 8 times with 10% HF. Finally, the soil samples were rinsed several times with distilled water and then dried at 60 °C and sieved with 60-mesh sieves before NMR measurements [30].

The soil microbial DNA was extracted with soil DNA kits (Omega Biotek, Norcross, GA, USA). We used a NanoDrop 2000 Spectrophotometer (NanoDrop Technologies, Wilmington, DE, USA) and 1% agarose gel electrophoresis to measure the DNA purity and concentration. All purified amplicons from all samples (V3–V4 region of bacteria; ITS region of fungi) were submitted to Majorbio BioPharm Technology Co., Ltd. (Shanghai, China) for paired-end sequencing on the Illumina HiSeq platform. The primers 515F (5′-GTGCCAGCMGCCGCGG-3′) and 806R (5′-GGACTACHVGGGTWTCTAAT-3′) were used to amplify bacteria. In addition, ITS1F (5′-CTTGGTCATTTAGAGGAAGTAA-3′) and ITS2R (5′-GCTGCGTTCTTCATCGATGC-3′) were used to amplify fungi. The PCR products were recovered and checked using gel electrophoresis and Axy Prep DNA Gel Extraction Kits (Axygen Biosciences, UnionCity, CA, USA).

#### 2.3.2. Plant Samples

The rice variety (J-Liangyou 1377) and wheat variety (Zhengmai 9023) were used in this experiment. Wheat samples were taken in summer (May 2019) and rice was harvested in autumn (September 2019). The plant nutrient concentrations were measured using an H_2_SO_4_–H_2_O_2_ digestion. We used an automated continuous flow analyzer to determine the plant N and P concentrations (AA3, Bran and Luebbe, Norderstedt, Germany). The potassium (K) content of the plant sample was assessed using a flame photometer (Flame Photometer PFP7, JENWAY, Staffordshire, UK).

### 2.4. Data Analysis

All data were expressed as mean ± standard deviation and calculated using Microsoft Excel (Microsoft Office, 2016). Statistical significance was evaluated via a one-way ANOVA with Duncan test (*p* < 0.05) or a two-way analysis of variance with SPSS (version 19.0). The RDA (vegan package), Mantel test (ggcor package) and two-factor network analyses were conducted to explore the relationship between the soil microbes and chemical properties with R software (version 4.2.3).

## 3. Results

### 3.1. Effects of Straw Return and Fertilization on Crop Yield and Nutrient Absorption

**Figure 1:**
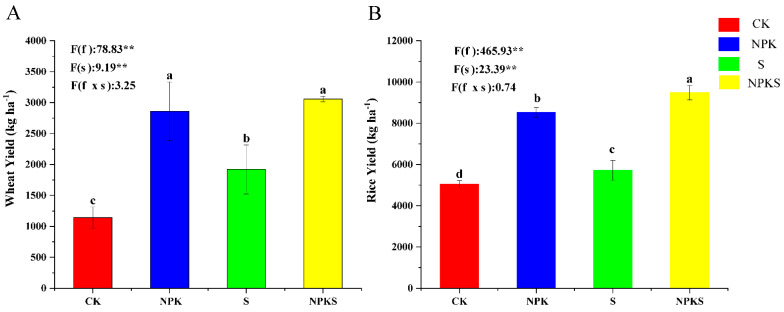
Wheat (**A**) and rice (**B**) crop yields across treatments. f: the main effect of fertilizer treatment; s: the main effect of straw treatment; f × s: the interaction effect of fertilizer and straw treatment. * Indicates significant difference at different levels (** *p* < 0.01). Different lowercase letter indicates significant differences according to Duncan’s test (*p* < 0.05).

The results showed that the wheat and rice yields increased under the NPKS treatment compared to CK. The yields of wheat and rice were increased from 1142.50 and 5045.45 kg ha^−1^ to 3057.50 and 9486.41 kg ha^−1^ under the NPKS treatment, representing an increase of 185.12% and 88.02%, respectively (Figure 1). In addition, it was found that the wheat and rice yields under the S treatment were significantly lower than those under the NPK treatment, representing a decrease of 32.87% and 33.01%, respectively (Figure 1). Two-factor ANOVA indicated that straw return and fertilization significantly affected the wheat and rice yield (*p* < 0.05) (Figure 1).

The N, P, and K contents in the crops were highest under the NPKS application in both wheat and rice, followed by the NPK, S, and CK treatment (Table 1). Compared to the CK treatment, the N, P, and K contents of the wheat stem were increased by 39.02%, 125%, and 20.23% under the NPKS treatment. However, the seed P content was significantly increased with straw return and the addition of fertilizer (Table 1). In addition, compared to the CK treatment, the N and K content of rice stems increased by 15.91% and 12.69%. Similarly, the K content of rice seed was significantly increased by 31.43% under the NPKS treatment (Table 1). Furthermore, the fertilization treatments significantly affected the N and P content of wheat (*p* < 0.05), and the straw return treatment significantly affected the K content of wheat and rice (Table 1).

### 3.2. Effects of Straw Return and Fertilization on Soil Fertility

The soil pH decreased by 0.26–0.48 units with chemical fertilizer (Table 2). Moreover, Table 2 shows that the SOM, TN, TP, AN, AP, AK, CEC, AFe, AMn, ACu, and AZn contents were increased by 49.12%, 32.62%, 35.06%, 22.89%, 129.36%, 48.34%, 13.40%, 133.95%, 58.98%, 18.26% and 33.33% under the NPKS treatment, respectively, compared to the CK treatment. Straw return influenced these metrics, and increased the SOM, TN, TP, AK, AMn, and AZn contents by 19.58%, 14.72%, 10.64%, 25.13%, 35.74%, and 27.66% with the NPKS treatment relative to chemical fertilizer alone (NPK) (Table 2). The main effects of straw had an obvious influence on soil fertility (*p* < 0.05) (Table 2).

**Figure 2:**
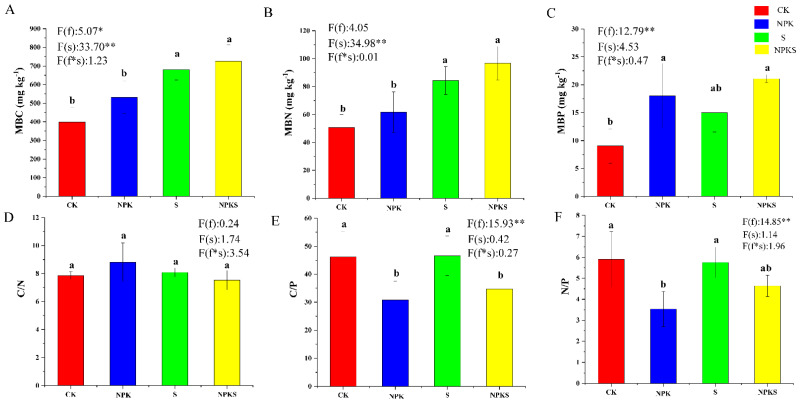
Microbial carbon (MBC), microbial nitrogen (MBN) and microbial phosphorus (MBP) under different treatments. (**A**–**C**): represent soil MBC, MBN and MBP, respectively; (**D**–**F**): represent the ratio of MBC/MBC, MBC/MBP and MBN/MBP. f: the main effect of fertilizer treatment; s: the main effect of straw treatment; f*s: the interaction effect of fertilizer and straw treatment. * Indicates significant difference at different levels (* *p* < 0.05, ** *p* < 0.01). Different lowercase letter indicates significant differences according to Duncan’s test (*p* < 0.05).

Compared to the CK treatment, MBC (Microbial Biomass Carbon), MBN (Microbial Biomass Nitrogen) and MBP (Microbial Biomass Phosphorus) were increased by 82.16%, 91.17%, and 133.18% under the NPKS treatment (Figure 2A–C). Furthermore, compared to conventional fertilizer application (NPK treatment), MBC and MBN were significantly higher under straw return (NPKS) by 36.38, 56.59% (Figure 2A,B). In addition, the ratios of C:P and N:P were decreased by 25.37–33.20% and 19.30–40.17% with K fertilizer application (Figure 2D,E). Moreover, the straw return treatments affected the ratio of C:P and N:P (Figure 2D,E).

### 3.3. Effects of Straw Return and Fertilization on Soil Aggregates

**Figure 3:**
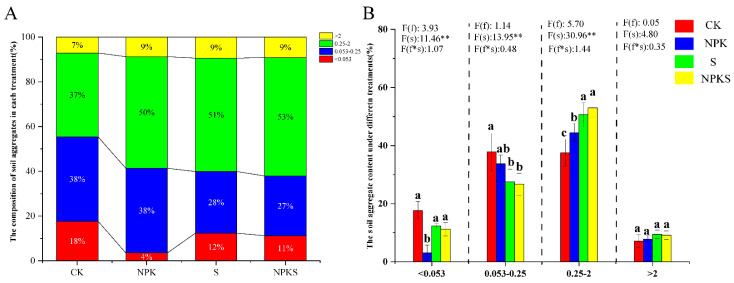
The content of soil aggregate under different treatments (**A**) and the composition of soil aggregate under different treatments (**B**). f: the main effect of fertilizer treatment; s: the main effect of straw treatment; f∗s: the interaction effect of fertilizer and straw treatment. * Indicates significant difference at different levels (** *p* < 0.01). Different lowercase letter indicates significant differences according to Duncan’s test (*p* < 0.05).

Fertilizer and straw return application impacted the distribution and relative abundance of soil aggregates (Figure 3A). The 0.25–2 mm aggregates were the main component, followed by 0.053–0.25, <0.053 and >2 mm accounting for 26.68–37.78%, 11.20–17.62%, and 7.11–9.95%, respectively (Figure 3A). Interestingly, the relative proportion of 0.25–2 mm aggregates increased with the addition of chemical fertilizer and straw. The size of 0.25–2 mm aggregates was increased from 37.49% to 52.97% under the NPKS treatment (Figure 3B). A reduction in aggregate size was observed in the 0.053–0.25 mm and <0.053 mm aggregates, decreasing from 33.78% and 17.62% (CK) to 26.68% and 11.20% in the NPKS treatment, respectively (Figure 3B). Straw return had a significant effect on four sizes of soil aggregates (Figure 3B). The results of the soil aggregate stability are shown in Figure S1. Compared to the CK treatment, NPKS addition increased the MWD, GMD, and R_0.25_ with fertilizer and straw return (Figure S1). The results showed that straw return and fertilizer application had a positive influence on the soil aggregate stability (Figure S1). NPKS addition reduced soil bulk by 6.40–12% compared to the CK treatment, though the soil capillary porosity increased by 10.02% under the NPKS treatment (Table S1).

### 3.4. Effects of Straw Return and Fertilizer Application on the Nutrients of Soil Aggregates

**Figure 4:**
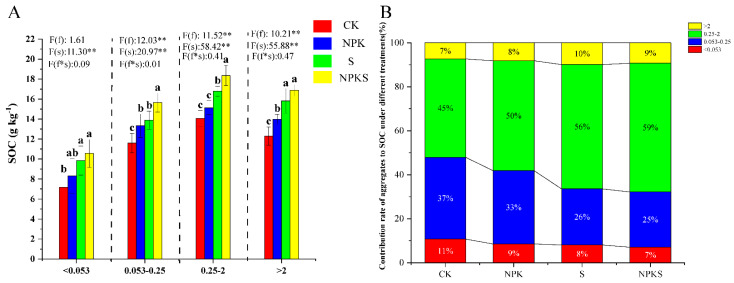
Soil organic carbon (SOC) in different soil aggregates under different treatments (**A**) and the relative contribution of each soil aggregate grouping to the total SOC under different treatments (**B**). f: the main effect of fertilizer treatment; s: the main effect of straw treatment; f*s: the interaction effect of fertilizer and straw treatments. * Indicates significant difference at different levels (** *p* < 0.01). Different lowercase letter indicates significant differences according to Duncan’s test (*p* < 0.05).

The SOC content was highest in the NPKS treatment, followed by the S and NPK treatments, and CK soils contained the least SOC (Figure 4). The SOC content of differently sized aggregates (<0.053 mm, 0.053–0.25 mm, 0.25–2 mm, >2 mm) was significantly increased under NPKS by 47.28, 34.83, 30.58 and 37.12, respectively, when compared to CK. Straw addition influenced the SOC content of 0.053–0.25, 0.25–2, and >2 mm soil aggregates, and the SOC was 17.33, 21.35, and 20.83% higher when comparing the NPKS and NPK treatment (Figure 4A). The study found that the highest contribution rate of soil organic carbon (SOC) was observed in the 0.25–2 mm aggregates (45–59%) across all treatments. This was followed by the 0.053–0.25 mm aggregates and the 2 mm aggregate soils showing the lowest SOC contribution rate (7–10%) (Figure 4B). Additionally, the SOC contribution rate in the 0.25–2 mm aggregates increased with the addition of straw and fertilizer, while the other three sizes of soil aggregates showed a decrease (Figure 4B).

Alkyl-C and O-alkyl-C were the main functional groups of SOC in the soil (Table 3). A detailed analysis revealed that different soil aggregates had different responses to straw addition (Table 3). For soil aggregates > 2 mm in size, the relative percent of alkyl-C and carbonyl C was increased, while the O-alkyl C was decreased (Table 3). However, the ratio of alkyl-C and O-alkyl C was decreased, while the aromatic C and carbonyl C content was promoted in other soil aggregates (Table 3).

**Figure 5:**
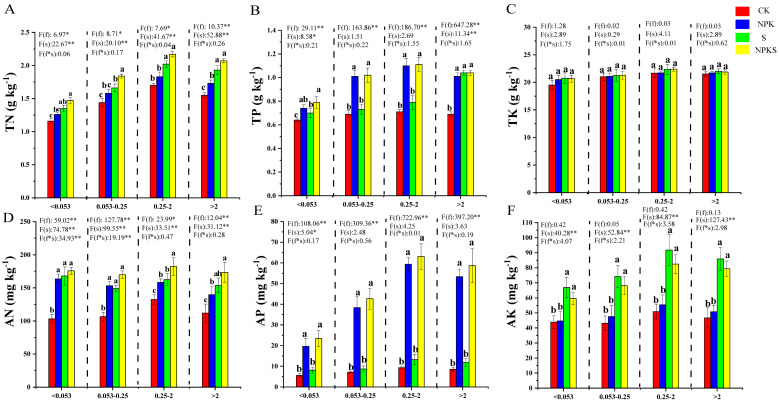
The relative contribution of each soil aggregate group to the total soil nutrient composition across treatments. (**A**–**F**): represents soil total nitrogen, total phosphorus, total potassium, available nitrogen, available phosphorus and available potassium. f: the main effect of fertilizer treatment; s: the main effect of straw treatment; f*s: the interaction effect of fertilizer and straw treatment. * Indicates significant difference at different levels (* *p* < 0.05, ** *p* < 0.01). Different lowercase letter indicates significant differences according to Duncan’s test (*p* < 0.05).

Compared to the CK treatment, the content of TN, TP, AN and AP increased under the NPKS treatment to different degrees in different soil aggregates (Figure 5). It was found that the fertilizer treatment significantly affected the soil P levels in different soil aggregates, whereas straw return had no significant effect on the soil P levels (except in the smallest and largest size fractions on TP level) (Figure 5B,E). It is noteworthy that the content of TK had no significant differences in the different soil aggregates among all treatments (Figure 5C). However, the soil AK was increased by 35.79%, 57.77%, 61.99%, and 70.04% in different soil aggregates under the NPKS treatment compared to the CK treatment (Figure 5E). Among all soil aggregates, the <0.053 mm aggregate had poor connections with soil nutrients compared to the other sizes of soil aggregate (Figure S2).

### 3.5. Effects of Straw Return and Fertilization on Soil Microorganisms

**Figure 6:**
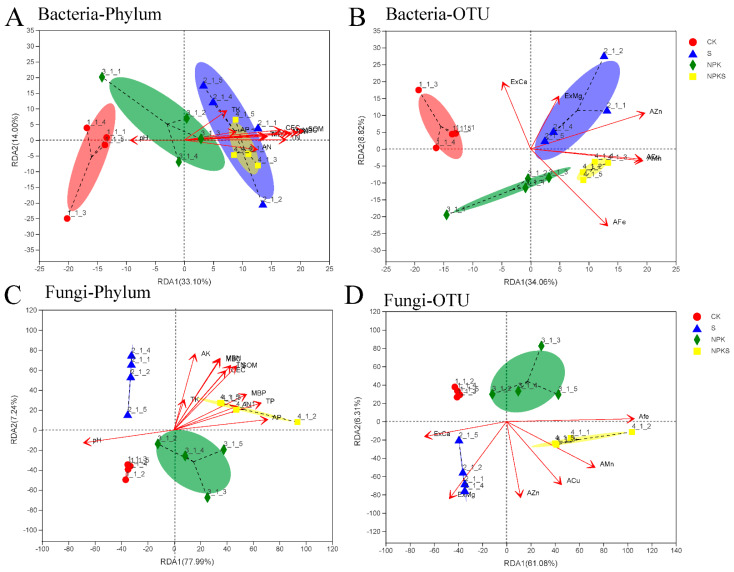
Reduction in RDA dimension for analyzing the microbial community structure across treatment types. (**A**,**B**): Phylum and OTU level of soil bacteria; (**C**,**D**): Phylum and OTU level of soil fungi.

The *α* diversity of bacteria was not affected by NPKS, although the *α* diversity of fungi was affected by the NPKS treatment compared to the CK treatment (Table S2). In addition, the community composition of bacteria on the phylum and the OTU level under the CK treatment were distinctly different from other treatments (straw and fertilizer) (Figure 6A,B). These differences in the community composition were more exaggerated in fungi than bacteria (Figure 6C,D). Moreover, the community composition of fungi was more sensitive to the changes in the soil environment, and soil had a stronger influence on soil fungi (Table S3).

**Figure 7:**
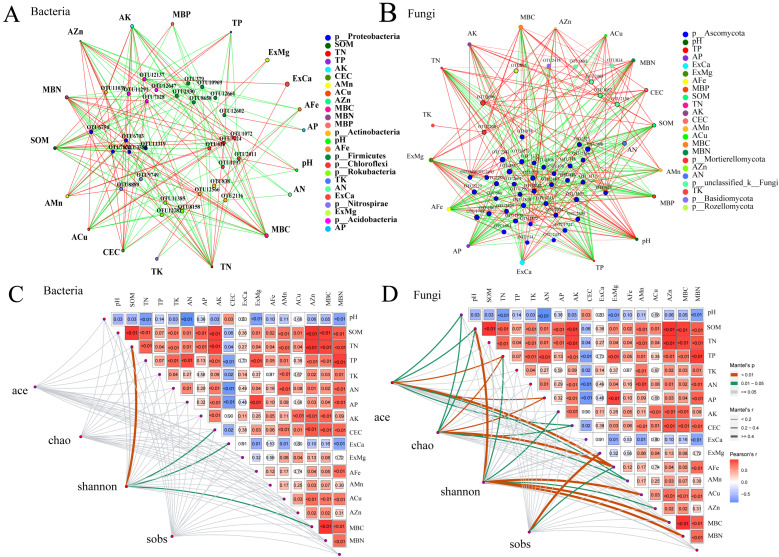
The two-factor network analysis (**A**,**B**) between soil microbes and chemical properties. (**A**,**B**): represents bacteria and fungi; the Mantel test (**C**,**D**) between soil microbes and chemical properties. (**C**,**D**): represents bacterial and fungi.

The effects of environmental factors on soil microorganisms were identified through a two-way network analysis (Figure 7A,B). In addition, the *p_Proteobacteria*, *p_Actinobacteria*, *p_Firmicutes*, *p_Chloroflexi*, *p_Rokubacteria*, *p_Nitrospirae* and *p_Acidobacteria* were sensitive bacteria to the soil environment (Figure 7A). The *p_Ascomycota*, *p_Mortierellomycota*, *p_Basidiomycota,* and *p_Rozellomycota* were important fungal microorganisms under different treatments (Figure 7B). There was a closer correlation between the fungal community and soil characteristics than the influence of soil on bacterial communities (Figure 7C,D). The bacterial alpha diversity indices (except for the Shannon index) were not closely related to soil properties (*p* > 0.05) (Figure 7C).

## 4. Discussion

### 4.1. Effects of Straw Return and Fertilization on the Growth of Crops

Many studies have shown that the addition of straw to soil can promote crop yields and improve nutrient uptake [31,32]. In our study, chemical fertilizer addition with straw return treatment was the best management strategy to improve crop yields (Figure 1). This could be a result of straw decomposition, which may increase soil nutrients [18,33]. In addition, straw mulching may improve soil water retention, potentially improving crop yields in arid and semi-arid farming areas [34,35]. Compared to conventional fertilization, straw mulching has increased wheat and rice yields (Figure 1) [36,37]. Straw application has increased the K content of wheat and rice, suggesting that K is easily released from straw as it decomposes [38]. In addition, the improvement effect of straw mulching is affected by many factors, such as the average annual temperature, soil nutrient status, period, and fertilization [39,40].

### 4.2. Effects of Straw Return and Fertilization on the Soil Physical Properties

Soil fertility reflects the soil’s capacity to supply nutrients for the growth of plants [41]. Many studies have reported that straw return improves soil fertility by promoting soil aggregation and the soil water retention capacity [42]. However, some studies have found that straw return decreases the amount of available N due to the high ratio of C/N [43]. Straw return application may also have effects on soil fertility [44,45]. In our study, the long-term application of straw return alone significantly improved soil fertility (Table 2). Several reasons support this finding. (1) Straw contains a variety of nutrients and improves soil fertility through decomposition, during which these nutrients are released and become available to plants [46]. (2) The input of straw provides C and N nutrients for soil microbial growth and reproduction, thus increasing the microbial community composition and diversity [47,48,49]. (3) Straw addition could influence the soil temperature and soil water retention capacity, thereby influencing the capacity for soil nutrient supply [50,51].

Soil aggregates can promote crop nutrient absorption, the diversity of biological communities, and reduce soil erosion by increasing the average size of aggregates [52,53,54,55]. Firstly, this could be due to a large aggregate turnover controlling the organic carbon stability in microaggregates [8]. Secondly, large aggregates have a high rate of organic carbon cycling and fresher organic carbon [56]. Finally, the higher organic carbon content within the macroaggregate protects and slows down the decomposition of organic carbon [57]. Our findings provide evidence that straw and fertilizer application could increase the proportion of soil aggregates (0.25–2 mm and >2 mm), while decreasing the proportion of other soil aggregates (0.053–0.025 mm and <0.053 mm) (Figure 3). There are several possible explanations for this result: (1) It could be that organic particles or colloids are generated by the application of straw to the field, which combines with the mineral particles in the soil to form micro-aggregates, and in turn provides a material basis for the formation of larger-sized aggregates [45]; (2) straw return may increase the activity of soil microorganisms, which secrete organic cementing substances or cement soil clay particles through the mycelium, thereby promoting the formation of aggregates [25]; and (3) during the process of straw decomposition, macromolecular mucopolysaccharides are produced, which strongly adhere to soil particles [58]. Therefore, straw application to the field can not only promote the formation of aggregates through direct action, but also indirectly by affecting microorganisms.

SOC is a significant indicator of soil fertility and productivity [59]. The creation of aggregates is an important mechanism for the physical protection of the SOC [21]. In our study, the SOC of all soil aggregates increased under the NPKS treatment (Figure 4). Zhang et al. [60] found that straw return was decomposed by soil microorganisms, which increased the content of various forms of carbon in the soil. Straw addition can promote the relative amount of aromatic C and carbonyl C groups (Table 3). Moreover, our results suggest that straw addition improved the soil capillary porosity (Table S2). This result is consistent with the work reported by Deng et al. [61]. The contribution rate of SOC in macro-aggregates increased while decreasing in micro-aggregates (Figure 4B). Based on a previous study, fertilizers can directly increase soil fertility [62]. Straw addition increased the nutrient concentrations of several different soil aggregates, particularly TN, AN, and AK (Figure 5). There were several reasons for these observations: (1) Straw addition may reduce soil N loss by improving soil water retention and soil texture [63]; and (2) straw addition is an important direct source of soil N and K [64] In our study, the straw had total N, P, and K contents of 0.54%, 0.09%, and 1.25%, respectively. Soil microbes can degrade organic compounds and alter inorganic products, especially contributing to the mineralization of nutrients [65]. Thus, straw addition may provide a food source for a variety of microorganisms that contribute to an overall ‘healthier’ environment [66]. Consequently, our results suggest that the combined application of straw and conventional fertilization could improve soil fertility and aggregate stability (Figure S1).

### 4.3. Effects of Straw Return and Fertilization on the Soil Microorganisms

Microorganisms are an important part of the soil environment, which can regulate the physical, chemical, and biological processes between soil and plants [67,68]. Different management measures affect the soil’s chemical properties and the microbial communities influenced [62,69]. For example, research has found that the soil pH, organic matter, and nutrient content all increase significantly after biochar application, which is considered to affect the soil microbial community structure [70]. Zhang et al. [71] found that the amounts of nitrifying bacteria, ammonizing bacteria and phosphorus-dissolving bacteria were increased with straw addition. Zhang et al. [72] found that the application of organic material could increase the amount of *p_Azotobacter* and *p_Pseudomonas*, which can improve K uptake. Moreover, straw addition increased the diversity and richness of microbial communities and changed the community composition (Figure 6). Straw has a positive impact on fungi, with the fungi community structure being more responsive to the addition of straw and fertilizer compared to the bacteria community (Figure 7; Table S2). When straw is incorporated into the field, fungi play an important role in decomposing recalcitrant fibers, which can be further decomposed into polysaccharides [73]. The initial decomposition of straw is mainly dominated by fungi and gram-negative bacteria, which influence soil microbial activity and nutrient cycling. Consequently, straw addition to the field is conducive to the accumulation of soil organic carbon and improving the abundance and activity of soil microbes [74]. Our study showed that a few microbial groups were particularly strongly associated with soil fertility, namely *p_Proteobacteria*, *p_Actinobacteria*, *p_Firmicutes p_Chloroflexi* and *p_Ascomycota* (Figure 7) [75,76].

## 5. Conclusions

In this study, wheat and rice yields significantly increased under NPKS addition after 14 years of rice–wheat rotation. Moreover, increases in the soil fertility of the soil (SOM, AN, AK) were largely attributed to straw addition. In addition, straw addition affected soil aggregation, particularly the formation of macro-aggregates (0.25–2 mm). Straw return also increased the content of SOC in several different sizes of soil aggregates. Furthermore, straw addition improved the soil nutrient content, particularly the soil SOC, TN, AN, and AK. The community structure of fungi was more susceptible to straw and fertilizer supplementation than soil bacteria. Thus, our present study suggested that straw addition could increase the soil fertility, soil aggregate stability, and crop yield, which become critical measures for increasing the utilization rate of agricultural waste for sustainable agricultural development.

## Figures and Tables

**Table 1:** The nutrients of wheat and rice under different treatments.

Treatment	Wheat						Rice					
	N (%)		P (%)		K (%)		N (%)		P (%)		K (%)	
	Stem	Seed	Stem	Seed	Stem	Seed	Stem	Seed	Stem	Seed	Stem	Seed
CK	0.41 ± 0.04 b	1.53 ± 0.09 a	0.04 ± 0.01 b	0.28 ± 0.02 b	0.84 ± 0.08 c	0.37 ± 0.02 b	0.44 ± 0.04 b	1.02 ± 0.09 ab	0.10 ± 0.01 a	0.29 ± 0.03 a	2.52 ± 0.09 b	0.35 ± 0.06 b
NPK	0.59 ± 0.09 a	1.61 ± 0.04 a	0.10 ± 0.02 a	0.37 ± 0.02 a	0.87 ± 0.04 bc	0.43 ± 0.03 a	0.45 ± 0.05 b	1.03 ± 0.06 ab	0.10 ± 0.01 a	0.29 ± 0.03 a	2.66 ± 0.08 ab	0.39 ± 0.0 3 ab
S	0.38 ± 0.07 b	1.49 ± 0.09 a	0.06 ± 0.01 b	0.35 ± 0.03 a	0.95 ± 0.08 ab	0.42 ± 0.03 ab	0.43 ± 0.03 b	1.00 ± 0.07 b	0.10 ± 0.01 a	0.31 ± 0.02 a	2.63 ± 0.09 b	0.42 ± 0.06 ab
NPKS	0.57 ± 0.16 a	1.59 ± 0.05 a	0.09 ± 0.02 a	0.35 ± 0.03 a	1.01 ± 0.03 a	0.41 ± 0.03 ab	0.51 ± 0.03 a	1.13 ± 0.06 a	0.11 ± 0.01 a	0.30 ± 0.02 a	2.84 ± 0.19 a	0.46 ± 0.08 a
f	12.92 **	5.98 *	35.36 **	10.42 **	2.2	2.62	4.64	4.01	0.12	0.24	9.02 *	2.12
s	0.24	0.70	0.17	3.07	14.38 **	0.77	2.21	1.44	1.27	0.97	5.58 *	4.74 *
f × s	0.01	0.02	5.86 *	13.78 **	0.31	4.82 *	2.83	2.86	1.34	0.01	0.44	0.01

Note: Mean ± Sd (n = 4). Different lowercase letters in a column indicate differences among treatments (*p* < 0.05). f: the main effect of fertilizer treatment; s: the main effect of straw treatment; f × s: the interaction effect of fertilizer and straw treatment. * Indicates significant difference at different levels (* *p* < 0.05, ** *p* < 0.01).

**Table 2:** The soil fertility under different treatments.

Treatment	pH	SOM (g kg^−1^)	TN (g kg^−1^)	TP (g kg^−1^)	TK (g kg^−1^)	AN (mg kg^−1^)	AP (mg kg^−1^)	AK (mg kg^−1^)	CEC (cmol kg^−1^)	Exch. Ca (mg kg^−1^)	Exch. Mg (mg kg^−1^)	Avail. Fe (mg kg^−1^)	Avail. Mn (mg kg^−1^)	Avail. Cu (mg kg^−1^)	Avail. Zn (mg kg^−1^)
CK	7.15 ± 0.10 a	17.69 ± 1.39 c	1.41 ± 0.17 c	0.77 ± 0.04 c	20.35 ± 0.27 a	119.17 ± 15.48 b	15.63 ± 3.37 b	56.89 ± 6.55 b	11.87 ± 0.54 b	3617.62 ± 599.62 a	315.31 ± 6.23 a	49.98 ± 10.60 b	21.38 ± 0.99 b	3.67 ± 0.31 b	0.45 ± 0.08 b
NPK	6.89 ± 0.28 ab	22.06 ± 0.53 b	1.63 ± 0.12 b	0.94 ± 0.05 b	20.56 ± 0.45 a	133.35 ± 13.81 ab	31.99 ± 7.53 a	67.44 ± 7.42 b	12.93 ± 0.46 a	2904.56 ± 328.21 a	297.09 ± 13.78 b	108.22 ± 16.31 a	25.04 ± 4.08 b	3.84 ± 0.38 ab	0.47 ± 0.08 b
S	7.11 ± 0.18 a	24.35 ± 1.55 a	1.76 ± 0.13 ab	0.84 ± 0.04 c	21.07 ± 0.61 a	130.22 ± 8.25 ab	19.59 ± 3.17 b	92.06 ± 9.98 a	13.21 ± 0.43 a	3510.14 ± 590.66 a	328.91 ± 10.36 a	67.92 ± 8.83 b	27.67 ± 4.76 ab	4.18 ± 0.27 ab	0.65 ± 0.09 a
NPKS	6.63 ± 0.20 b	26.38 ± 1.64 a	1.87 ± 0.12 a	1.04 ± 0.08 a	20.99 ± 0.59 a	146.45 ± 14.88 a	35.85 ± 4.91 a	84.39 ± 6.80 a	13.46 ± 0.21 a	2881.99 ± 332.69 a	314.38 ± 9.55 a	116.93 ± 16.57 a	33.99 ± 6.81 a	4.34 ± 0.36 a	0.60 ± 0.06 a
f	13.80 **	22.29 **	5.60 *	44.17 **	0.15	5.13 *	41.62 **	0.13	9.10 *	7.76 *	10.05 **	62.94 **	4.58 **	0.94	0.11
s	2.27	65.91 **	18.08 **	9.99 **	0.98	3.24	2.39	44.55 **	19.08 **	0.07	8.93 *	3.88	10.72 **	9.21 *	19.23 **
f × s	1.22	3.00	0.77	0.15	0.07	0.02	0.01	5.44 *	3.61	0.03	0.13	0.47	0.33	0.01	0.69

Note: Mean ± Sd (n = 4) for a series of fertility measurements. Different lowercase letters in a column indicate differences among treatments (*p* < 0.05), f: the main effect of fertilizer treatment; s: the main effect of straw treatment; f × s: the interaction effect of fertilizer and straw treatment. * Indicates significant difference at different levels (* *p* < 0.05, ** *p* < 0.01). The SOM, TN, TP, TK, AN, AP, AK, CEC, exchangeable Ca, exchangeable Mg, available Fe, available Mn, available Cu and available Zn: soil organic matter, total nitrogen, total phosphorus, total Potassium, available nitrogen, available phosphorus, available potassium, exchangeable cation, exchangeable calcium, exchangeable magnesium, available iron, available manganese, available copper and available zinc.

**Table 3:** Relative content of organic carbon components in soil aggregates under different treatments.

Treatment		Alkyl C	O-Alkyl C				Aromatic C	Carbonyl C
		0–45 ppm	45–60 ppm	60–95 ppm	95–110 ppm	Total	110–160 ppm	160–220 ppm
		CH_3_/CH_2_	OCH_3_/NCH	Carbohydrate	O-C-O		C=O/Aryl	COO/NC=O
>2	CK	23.46	19.59	51.75	3.38	74.71	1.77	0.05
	NPK	23.61	10.54	45.23	10.90	66.68	9.71	0.01
	S	36.78	16.83	35.85	6.67	59.35	3.08	0.79
	NPKS	37.03	16.64	39.32	5.12	61.08	1.46	0.42
0.25–2	CK	25.57	13.64	34.00	7.36	55.00	11.79	7.64
	NPK	28.80	16.76	41.73	4.96	63.44	3.33	4.42
	S	23.25	11.08	33.67	6.75	51.50	17.00	8.25
	NPKS	23.00	12.00	30.10	7.10	49.20	17.40	10.40
0.05–0.25	CK	26.95	11.21	31.89	6.87	49.97	14.14	8.94
	NPK	24.67	11.87	35.47	6.93	54.27	14.13	6.93
	S	22.69	12.38	32.92	7.38	52.69	16.00	8.62
	NPKS	20.58	11.39	32.27	7.69	51.35	16.78	11.29
<0.05	CK	30.65	13.85	31.65	5.42	50.92	9.32	9.11
	NPK	34.67	13.76	27.14	4.86	45.76	10.00	9.57
	S	21.52	9.68	31.36	6.99	48.04	16.60	13.84
	NPKS	25.13	11.00	34.00	6.87	51.87	12.47	10.53

Note: Colored cells denote ‘heatmap’ visualization of the differences between treatments with column means, where green indicates values below the respective means and red indicates values above the respective means.

## Data Availability

Data are contained within the article and supplementary materials.

## Supplementary Materials

The following supporting information can be downloaded at: https://www.mdpi.com/article/10.3390/plants13070985/s1, Figure S1: The stability of soil aggregates under different treatments.; Figure S2: The relationship between soil fertility and soil nutrients of different aggregate. (A–D): represented the different soil aggregate (<0.053 mm; 0.053–0.25 mm; 0.25–2 mm; >2 mm); Table S1: The bulk density and soil porosity under different treatments; Table S2: The α diversity of soil bacterial and fungal community under different treatments; Table S3: The datasheet for environmental factors in RDA.
