# Supplementary material for: Straw Addition Enhances Crop Yield, Soil Aggregation, and Soil Microorganisms in a 14-Year Wheat–Rice Rotation System in Central China
Source: Plants (Basel). 2024 Mar 29;13(7):985. doi: 10.3390/plants13070985 (PMC11013638; doi:10.3390/plants13070985)
Supplement: Supplementary file 1 [file plants-13-00985-s001.zip › plants-2860905-supplementary.pdf]

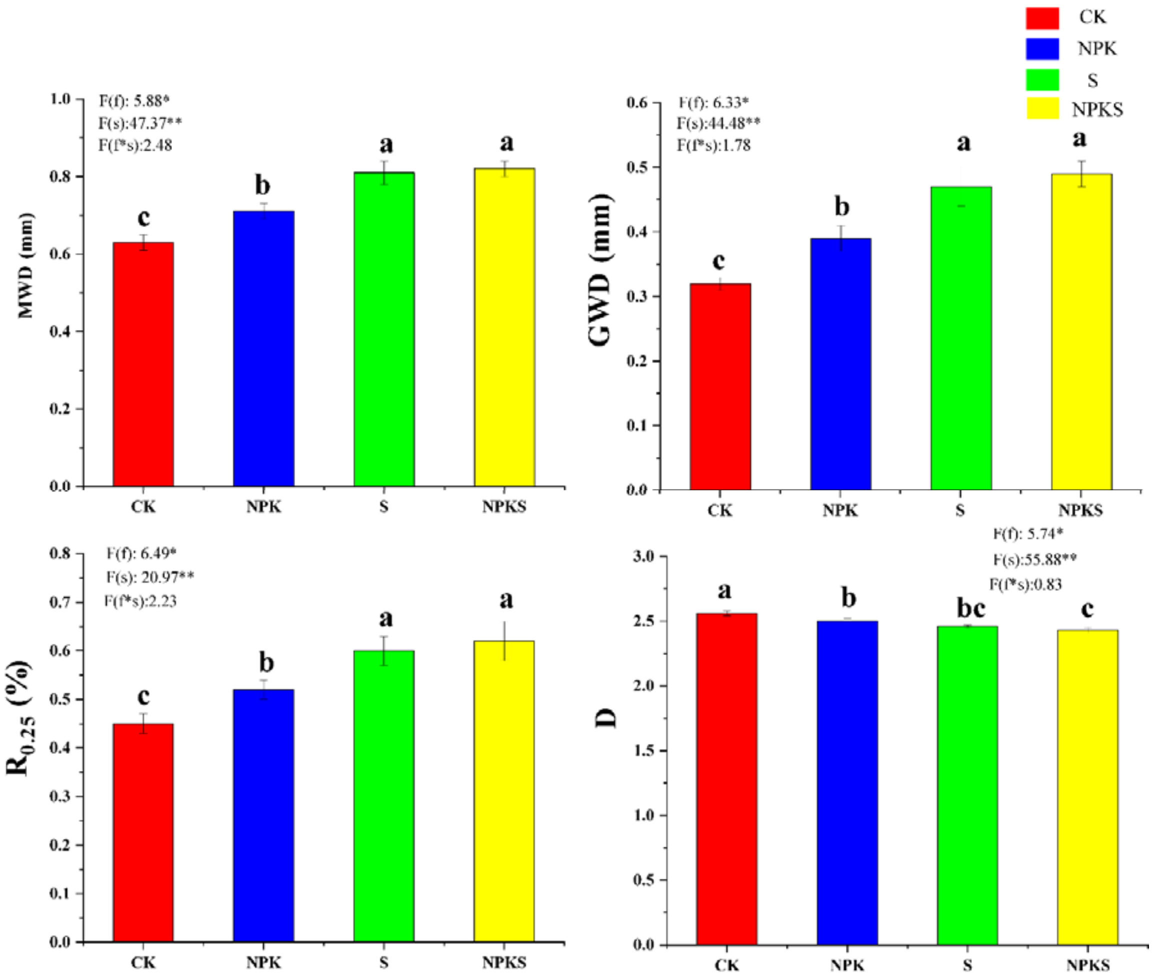

Figure S1. The stability of soil aggregates under different treatments. * Indicates significant difference at different levels (* $p < 0.05$, ** $p < 0.01$). Different lowercase letter indicates significant differences according to Duncan's test ($p < 0.05$).

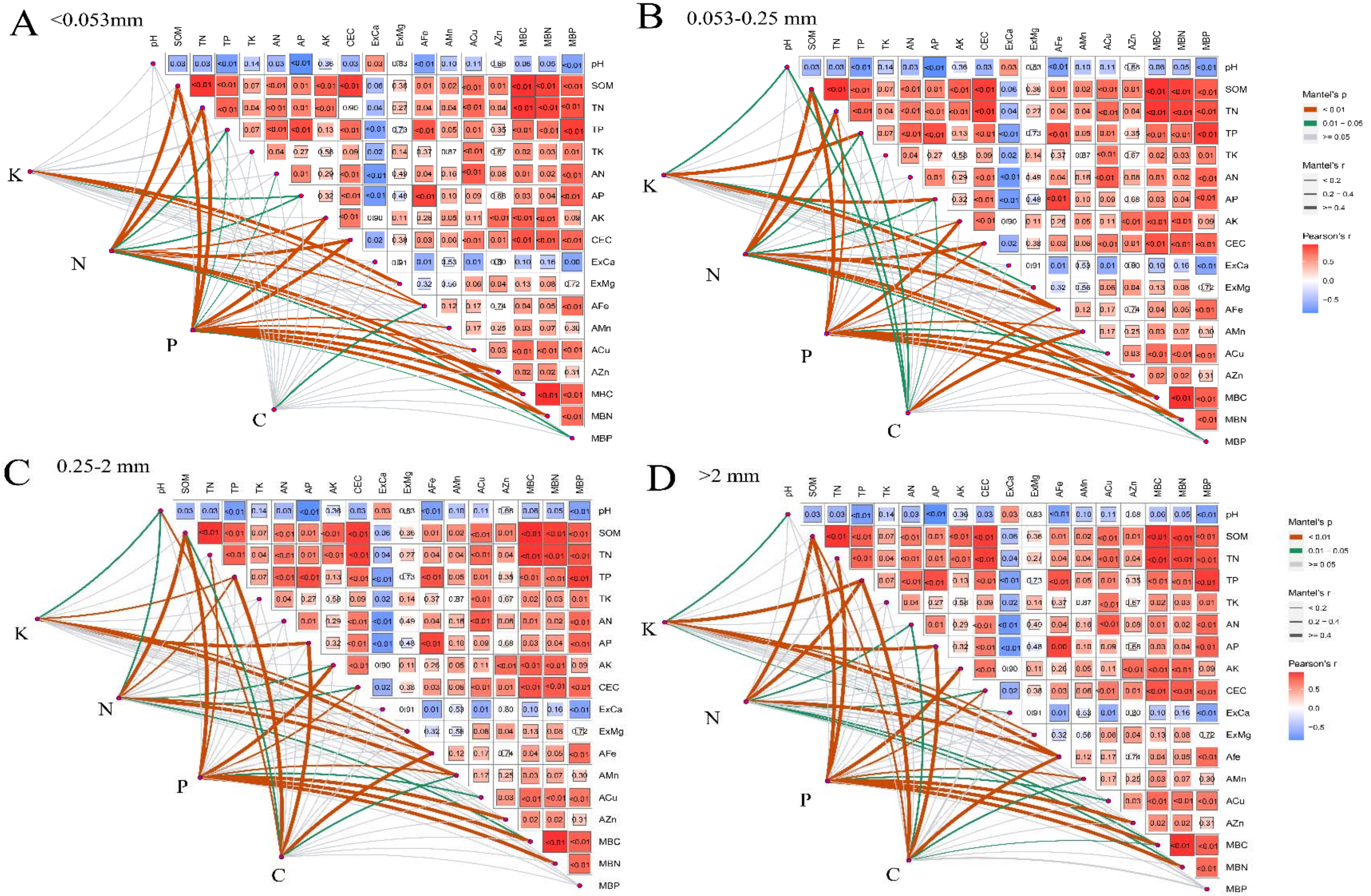

Figure S2. The relationship between soil fertility and soil nutrients of different aggregate. A-D: represented the different soil aggregate (< 0.053mm; 0.053-0.25mm; 0.25-2mm; > 2mm).

Table S1. The bulk density and soil porosity under different treatments. Different lowercase letters in a column indicate differences among treatments ($p < 0.05$).

Treatment	Bulk density (g cm^{-3})	Soil capillary porosity (%)	Soil non-capillary porosity (%)
CK	1.25±0.02a	48.21±1.66b	4.80±2.14a
NPK	1.17±0.03b	50.64±2.47ab	5.19±2.95a
S	1.11±0.05b	53.22±1.36a	4.92±1.27a
NPKS	1.10±0.06b	53.04±2.92a	5.47±1.86a

Table S2. The α diversity of soil bacterial and fungal community under different treatments. Different lowercase letters in a column indicate differences among treatments ($p < 0.05$).

Treatment	Bacteria				Fungi			
	sobs	shannon	ace	chao	sobs	shannon	ace	chao
CK	3978.20±78.11a	7.19±0.07a	7330.10±129.45ab	6108.60±81.15a	561.50±39.14b	4.50±0.12a	587.20±38.15c	593.10±38.37b
NPK	4006.00±175.81a	7.26±0.08a	7015.80±414.32b	6101.80±161.90a	568.25±33.34b	3.81±0.30b	666.26±86.76bc	658.73±77.12b
S	3974.80±130.75a	7.24±0.08a	7303.20±384.34ab	6034.00±300.58a	693.00±48.68a	4.47±0.04a	753.68±57.40ab	758.33±56.14a
NPKS	4086.00±69.68a	7.26±0.04a	7507.30±117.14a	6243.50±31.47a	632.25±39.98a	3.67±0.46b	769.98±58.32a	763.73±53.71a

Table S3. The datasheet for environmental factors in RDA.

Bacteria	RDA1	RDA2	r2	p_values	Fungi	RDA1	RDA2	r2	p_values
pH	-0.999	-0.040	0.134	0.389	pH	-0.985	-0.172	0.659	**0.001**
SOM	0.986	0.167	0.707	**0.001**	SOM	0.623	0.782	0.824	**0.001**
TN	0.999	0.041	0.498	**0.009**	TN	0.583	0.813	0.773	**0.001**
TP	0.993	0.117	0.315	0.096	TP	0.935	0.354	0.686	**0.003**
TK	0.638	0.771	0.221	0.213	TK	0.267	0.964	0.113	0.428
AN	0.982	-0.191	0.261	0.164	AN	0.909	0.418	0.378	**0.037**
AP	0.953	0.302	0.135	0.397	AP	0.991	0.135	0.701	**0.002**
AK	0.991	0.138	0.565	**0.003**	AK	0.219	0.976	0.759	**0.001**
CEC	0.976	0.217	0.470	**0.015**	CEC	0.579	0.815	0.647	**0.001**
MBC	0.989	0.151	0.633	**0.003**	MBC	0.471	0.882	0.792	**0.001**
MBN	0.989	0.150	0.531	**0.010**	MBN	0.465	0.886	0.812	**0.001**
MBP	0.994	0.111	0.332	0.088	MBP	0.857	0.516	0.568	**0.005**
ExCa	-0.277	0.961	0.461	**0.015**	ExCa	-0.982	-0.187	0.281	0.129
ExMg	0.269	0.963	0.281	0.118	ExMg	-0.429	-0.903	0.555	**0.005**
AFe	0.530	-0.848	0.808	**0.001**	AFe	0.999	-0.042	0.673	**0.001**
AMn	0.977	-0.213	0.447	**0.025**	AMn	0.793	-0.610	0.515	**0.008**
ACu	0.982	-0.192	0.434	**0.025**	ACu	0.547	-0.837	0.453	**0.022**
AZn	0.896	0.445	0.549	**0.005**	AZn	0.192	-0.981	0.453	**0.025**
